# Shared and Distinct Genomics of Chronic Thromboembolic Pulmonary Hypertension and Pulmonary Embolism

**DOI:** 10.1164/rccm.202307-1236OC

**Published:** 2024-03-12

**Authors:** James Liley, Michael Newnham, Marta Bleda, Katherine Bunclark, William Auger, Joan Albert Barbera, Harm Bogaard, Marion Delcroix, Timothy M. Fernandes, Luke Howard, David Jenkins, Irene Lang, Eckhard Mayer, Chris Rhodes, Michael Simpson, Laura Southgate, Richard Trembath, John Wharton, Martin R. Wilkins, Stefan Gräf, Nicholas Morrell, Joanna Pepke Zaba, Mark Toshner

**Affiliations:** ^1^ Durham University, Durham, United Kingdom;; ^2^ Institute of Applied Health Research, Birmingham, United Kingdom;; ^3^ Department of Medicine, University of Cambridge, Cambridge, United Kingdom;; ^4^ Royal Papworth Hospital, Cambridge, United Kingdom;; ^5^ University of California, San Diego, San Diego, California;; ^6^ Hospital Clinic, Institut d’Investigacions Biomèdiques August Pi i Sunyer, Centro de Investigación Biomédica en Red Enfermedades Respiratorias, University of Barcelona, Barcelona, Spain;; ^7^ Amsterdam University Medical Center, Amsterdam, the Netherlands;; ^8^ University Hospitals Leuven, Leuven, Belgium;; ^9^ Hammersmith Hospital, London, United Kingdom;; ^10^ Vienna General Hospital, Medical University of Vienna, Vienna, Austria;; ^11^ Kerckhoff Clinic, Bad Nauheim, Germany;; ^12^ Imperial College London, London, United Kingdom;; ^13^ King’s College London, London, United Kingdom; and; ^14^ St. George’s, University of London, London, United Kingdom

**Keywords:** genome-wide association study, pulmonary arterial hypertension, venous thromboembolism

## Abstract

**Rationale:**

Chronic thromboembolic pulmonary hypertension involves the formation and nonresolution of thrombus, dysregulated inflammation, angiogenesis, and the development of a small-vessel vasculopathy.

**Objectives:**

We aimed to establish the genetic basis of chronic thromboembolic pulmonary hypertension to gain insight into its pathophysiological contributors.

**Methods:**

We conducted a genome-wide association study on 1,907 European cases and 10,363 European control subjects. We coanalyzed our results with existing results from genome-wide association studies on deep vein thrombosis, pulmonary embolism, and idiopathic pulmonary arterial hypertension.

**Measurements and Main Results:**

Our primary association study revealed genetic associations at the ABO, FGG, F11, MYH7B, and HLA-DRA loci. Through our coanalysis, we demonstrate further associations with chronic thromboembolic pulmonary hypertension at the F2, TSPAN15, SLC44A2, and F5 loci but find no statistically significant associations shared with idiopathic pulmonary arterial hypertension.

**Conclusions:**

Chronic thromboembolic pulmonary hypertension is a partially heritable polygenic disease, with related though distinct genetic associations with pulmonary embolism and deep vein thrombosis.

**At a Glance Commentary**

**Scientific Knowledge on the Subject**

Chronic thromboembolic pulmonary hypertension is a respiratory illness characterized by many potential pathophysiological processes, including formation and nonresolution of thrombus, dysregulated inflammation, angiogenesis, vasculopathy, and right heart failure. There are no previous investigations of its genetic causes on a genome-wide scale, and, by understanding the genetic contributors to disease, it is likely we will better understand the pathophysiology.

**What This Study Adds to the Field**

We conduct the first genome-wide association study on chronic thromboembolic pulmonary hypertension. We find several regional genetic associations and partial sharing of genetic associations with pulmonary embolism. We do not find evidence of shared genetics with idiopathic pulmonary arterial hypertension.

Chronic thromboembolic pulmonary hypertension (CTEPH) is characterized by the organization and fibrosis of thromboembolic material leading to the obstruction of proximal pulmonary arteries, which, together with a secondary small-vessel vasculopathy, results in pulmonary hypertension and subsequent right heart failure.

CTEPH is conventionally considered to result from a process of disordered thrombus resolution after one or more episodes of acute pulmonary embolism (PE) (1). The pathobiology of thrombus nonresolution after acute PE, however, remains poorly understood but likely arises from complex interactions between mediators of the coagulation cascade, angiogenesis, platelet function, and inflammation in association with host factors. Large-volume acute PEs, idiopathic presentation, and PE recurrence are associated with a risk for CTEPH development (2). Inefficient anticoagulation may also trigger thrombus formation (3). These factors, however, do not serve to explain the development of CTEPH in most patients. Furthermore, up to 25% of patients with CTEPH do not have a history of antecedent PE. The ability to identify abnormalities in coagulation/fibrinolysis pathways in patients with CTEPH is compounded by their treatment with therapeutic anticoagulation and lack of a good animal model of CTEPH.

Genetic studies in CTEPH have the potential to inform our understanding of disease pathophysiology but have thus far been hampered by the challenge of assembling cases in rare diseases. A European prospective registry found an increased CTEPH risk in non-O blood groups in a similar pattern to deep vein thrombosis (DVT) and PE (4), indicating a genetic association with the disease at this locus. This differential risk with ABO is also seen in overall risk of PE and other clotting disorders. To our knowledge, no other genetic associations with CTEPH have been confirmed at genome-wide significance (*P* < 5 × 10^−8^).

The genetic basis of a comparator disease, idiopathic pulmonary arterial hypertension (IPAH), has been explored much more systematically. Heterozygous germline mutations in *BMPR2* are found in 10–20% of individuals with IPAH alongside rarer sequence variants, including *SMAD9*, *ACVRL1*, *ENG*, *KCNK3*, and *TBX4* (5). A more recent genome-wide association study (GWAS) has also identified common variants contributing to IPAH etiology and clinical course (6).

An improved understanding of the genetic basis of CTEPH has the potential not only to inform disease etiopathogenesis but also to quantify CTEPH risk and develop preventative strategies and treatment options. An evaluation of CTEPH genome-wide associations is therefore warranted. Coanalysis with existing GWASs in PE and DVT aims to improve both discovery and the interpretation of results in comparison with other venous thromboembolic phenotypes. Given well-known genetic drivers of the development of IPAH and its shared pathobiological features of vascular remodeling, inflammation, and dysregulated angiogenesis with CTEPH, genetic associations between CTEPH and IPAH were also explored. Some of the results of these studies were previously reported in the form of a preprint (www.medrxiv.org/content/10.1101/2023.05.30.23290666v2).

## Methods

### Study Samples and Participants

The study was approved by the regional ethics committee (REC nos. 08/H0802/32 and 08/H0304/56). All study participants provided written informed consent from their respective institutions.

### GWAS on CTEPH

We conducted a study with a two-stage design: a discovery study including only U.K. samples and a replication stage using non-U.K. cases and a mixture of non-U.K. and U.K. control subjects. CTEPH was diagnosed in accordance with international guidelines (7). All patients were diagnosed through internationally accredited specialist centers with multimodal imaging and invasive hemodynamics. The U.K. cases, in addition to review at nationally designated tertiary center multidisciplinary teams, are additionally reviewed at the national CTEPH multidisciplinary team where all cases are discussed by a multidisciplinary team of surgeons, cardiologists, radiologists, and pulmonary hypertension specialists. Demographics of CTEPH samples are reported in Table E1 in the online supplement. Consistent with historical published cohorts (4), pulmonary endarterectomy treatment was most common (62.4%), followed by balloon pulmonary angioplasty (2.4%), both pulmonary endarterectomy and balloon pulmonary angioplasty (1.6%), and medical therapy only (33.6%). Control subjects were sourced randomly from the population (without requiring absence of thromboembolic phenotypes). Samples in the discovery phase were genotyped on one of four platforms: the Illumina HumanOmniExpress Exome-8 version 1.2 BeadChip (1,555 cases, 1,693 control subjects), the Illumina HumanOmniExpressExome-8 version 1.6 BeadChip (372 cases, 12 control subjects), the Affymetrix Axiom Genome-Wide CEU 1 Array (541 cases, 5,984 control subjects, including regenotyping of 1,533 control subjects genotyped on the Illumina HumanOmniExpressExome-8 version 1.2 BeadChip), and the Affymetrix UK Biobank Axiom array (6,717 control subjects).

We performed sample- and SNP-wise quality control on our dataset (8) and excluded cases of non-European ancestry using principal components generated using the 1000 Genomes Project. We imputed all genotypes to whole-genome cover using the Haplotype Reference Consortium panel on the Sanger imputation server (9, 10), separating samples by genotyping platform, and we included SNPs with an INFO score of at least 0.5 across all genotyping platforms used in the study. The INFO score is a measure of imputation accuracy, interpretable as a proportion: a score of 1 indicates full knowledge of the SNP in all samples, 0 indicates no knowledge of the SNP, and other values indicate knowledge of the SNP equivalent to full knowledge in that proportion of samples (11). Full details of quality control procedures are provided in the Methods section of the online supplement.

We separated the discovery cohort into two groups by genotyping platform (Affymetrix or Illumina) and analyzed each separately. In each cohort, we used a logistic regression with 10 principal component covariates to generate association statistics and corrected results for residual genomic inflation (12). Because each analysis involved separate samples, we combined results across platforms using a routine *P* value meta-analysis using Fisher’s method accounting for effect directions.

### Coanalysis with DVT and PE

To enhance our power to detect CTEPH associations, we coanalyzed our *P* values from the CTEPH meta-analysis with *P* values derived from GWAS on self-reported PE and DVT drawn from the UK Biobank (13) (GWAS round 2; self-reported DVT [code 20002_1094] and self-reported PE [code 20002_1093]). Details of the coanalysis are provided in the Methods section of the online supplement. In short, the output of each coanalysis is a set of *P* values for CTEPH “adjusted” for the overall genetic similarity between CTEPH and the second disease (14), which we call “V-values.” We also performed an analysis using results from DVT in place of results from PE, but we found the results from the two analyses were very similar, so we focus principally on the analysis of PE.

### CTEPH GWAS Associations

Noting that our replication cohort was analyzed at genome-wide SNPs, we defined genetic associations at three tiers of significance, all of which generally correspond to a genome-wide significance of overall *P* value <5 × 10^−8^ with varying levels of evidence in the discovery and replication subcohorts. The first tier required *P* < 5 × 10^−6^ in the combined discovery cohort, *P* < 5 × 10^−3^ in the replication cohort, and *P* < 5 × 10^−8^ in the combined meta-analysis, with consistent directions of effect across the two subanalyses in the discovery study and in the replication study. The second tier, designed to ensure nominal association in each cohort and overall genome-wide significance, required a nominal association of *P* < 5 × 10^−2^ in discovery and replication cohorts and *P* < 5 × 10^−8^ in the overall meta-analysis, again with consistent directions of effect. The “adjusted” *P* values allowed a comparison of evidence for association using conditional false discovery rate in a similar way to a comparison using meta-analyzed *P* values, and hence we defined a third tier of association requiring a *P* value of 5 × 10^−8^ in either the overall meta-analysis or the “adjusted” sets of *P* values derived from leverage of the CTEPH summary statistics on summary statistics for PE, together with consistent directions of effect in discovery and replication cohorts. All *P* value thresholds used in “tier” definitions were chosen before observing the data.

**Figure 1.**
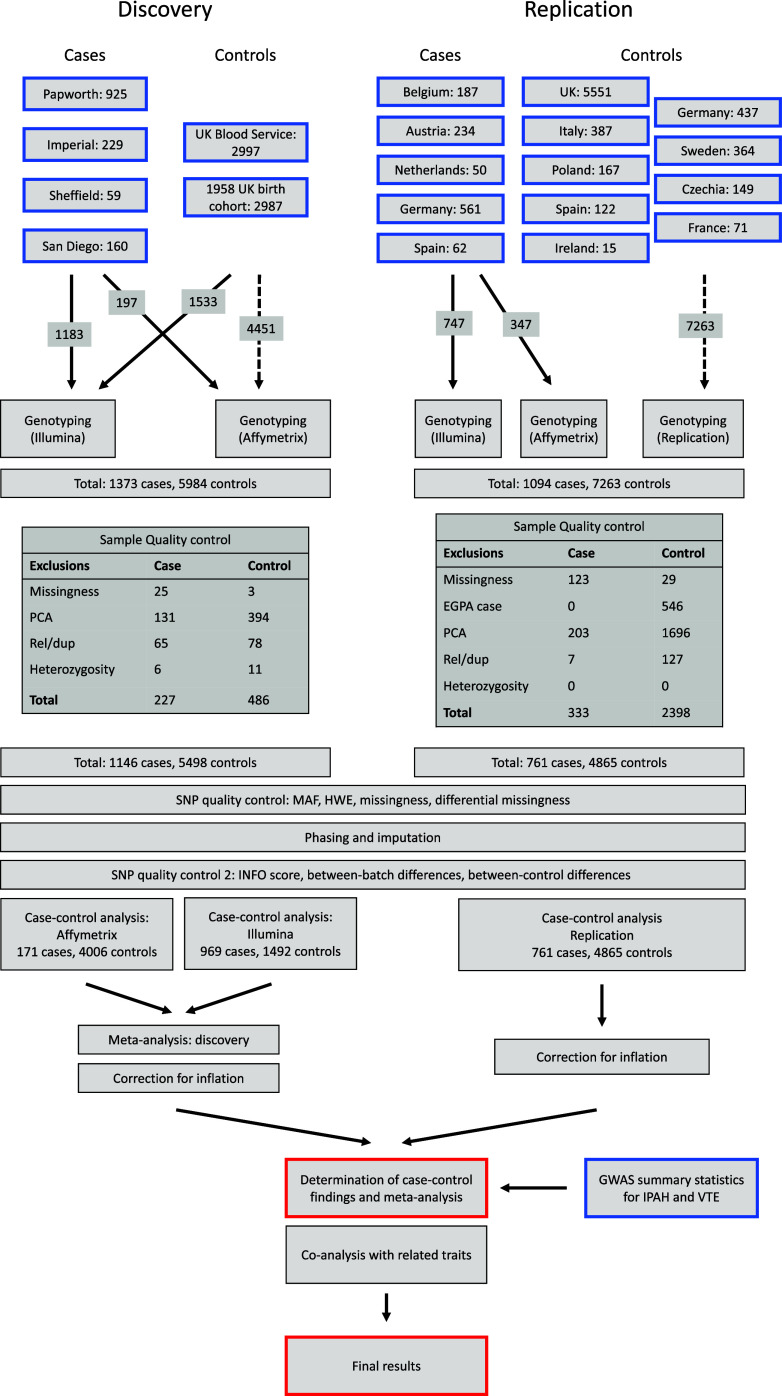
Flowchart of the study design. Principal component analysis (PCA): excluded due to inferred ancestry based on PCA. Rel/dup: excluded due to being closely related to another sample, or a duplicate of another sample. Heterozygosity: excluded due to abnormal heterozygosity rate. Missingness: excluded due to high missingness in genotype or otherwise unusable genotype. Full details are provided in the Methods section of the online supplement. In short, we recruited cases and control subjects from a variety of centers around the United Kingdom and Europe, including existing control subjects. Our discovery cohort consisted of U.K. and U.S. samples; our replication cohort consisted of European samples. Exclusions were applied sequentially: For instance, some samples that would have been excluded for abnormal heterozygosity rate in the replication cohort were already excluded by PCA. EGPA = eosinophilic granulomatosis with polyangiitis; GWAS = genome-wide association study; HWE = Hardy-Weinberg equilibrium; IPAH = idiopathic pulmonary arterial hypertension; MAF = minor allele frequency; VTE = venous thromboembolism.

There was a distribution of cases and control subjects across genotyping batches that could enable confounding batch effects, and differing sources of cases and control subjects in the replication cohort necessitated across-platform comparisons and imperfect geographical matching, resulting in high inflation in association statistics. We also noted recent work indicating that blood bank–sourced control samples may have distributions of ABO blood groups differing from the general population, potentially biasing association statistics at that locus. In the online supplement, we analyze allele frequencies across batches and cohorts directly and thus demonstrate that these confounding effects are unlikely to drive our positive associations. The study design is outlined in Figure 1.

### Genetic Overlap with IPAH and PE

We considered as a cause of pulmonary arterial hypertension the possibility that CTEPH shares pathology with IPAH. We first assessed whether our findings had any associations in common with a recent GWAS on IPAH (6). To assess for genome-scale similarity in genetic basis between IPAH and CTEPH, we used linkage disequilibrium score regression (15) to estimate genetic correlation *ρ_g_* between the two traits, which measures the degree of shared genetic basis. We also estimated genetic correlation between IPAH and PE (using the summary statistics for PE used in the coanalysis with CTEPH) for comparison.

Diseases with identical genetic bases have genetic correlation 1, and diseases with completely independent genetic bases have genetic correlation 0. If IPAH and CTEPH each occurred as a consequence of some identical underlying cause, we would expect them to have genetic correlation 1, whereas if they were caused by completely independent pathological processes, the genetic correlation would be 0 (and likewise for PE and CTEPH).

### Comparison of DVT, PE, and CTEPH

We would expect to see a slight difference in observed effect sizes between CTEPH, PE, and DVT at any given variant due to random variation across studies. For each of our CTEPH-associated variants, we assessed whether the observed effect sizes in CTEPH and PE were consistent with random variation if CTEPH and PE/DVT had identical underlying genetic causes. Specifically, we considered a null hypothesis that the two diseases have identical effect sizes for all SNPs, and we assessed the probability of seeing large differences in effect sizes between CTEPH and PE. Our approach is detailed in the Methods section of the online supplement, subsection “Differential Effect Sizes between CTEPH, DVT and PE.”

## Results

### GWAS on CTEPH

After quality control (*see* Figure 1), our dataset consisted of 1,146 cases and 5,498 control subjects in the discovery cohort and 761 cases and 4,865 control subjects in the replication cohort. A total of 4,655,481 SNPs passed quality control and were included in the final analysis. At tier 2 significance, the study had approximately 80% power to detect an odds ratio of 1.3 for an SNP of minor allele frequency 0.25 or an odds ratio of 1.7 for an SNP of minor allele frequency 0.05. Further details of power for tiers 1 and 2 significance are shown in Figures E1–E3. Minimal detectable effect sizes at tier 3 significance are more complex; *see* the Methods section of the online supplement.

We computed genomic inflation factors (λ) that measure the overall distribution of *P* values and should be close to 1. These were 0.95 in the discovery cohort and 1.21 in the replication cohort (λ_1000_ = 1.16; *see* the Methods section in the online supplement [16]), suggesting that *P* values in the replication cohort were overall lower than expected. We were not able to reduce inflation in the replication cohort by inclusion of further covariates or by use of linear mixed models, and we concluded that the degree of inflation was inevitable, given the imperfect geographical matching between cases and control subjects in the replication dataset. We corrected *P* values in the replication cohort for this residual inflation (12), thereby avoiding false-positive results arising from this inflation.

**Figure 2.**
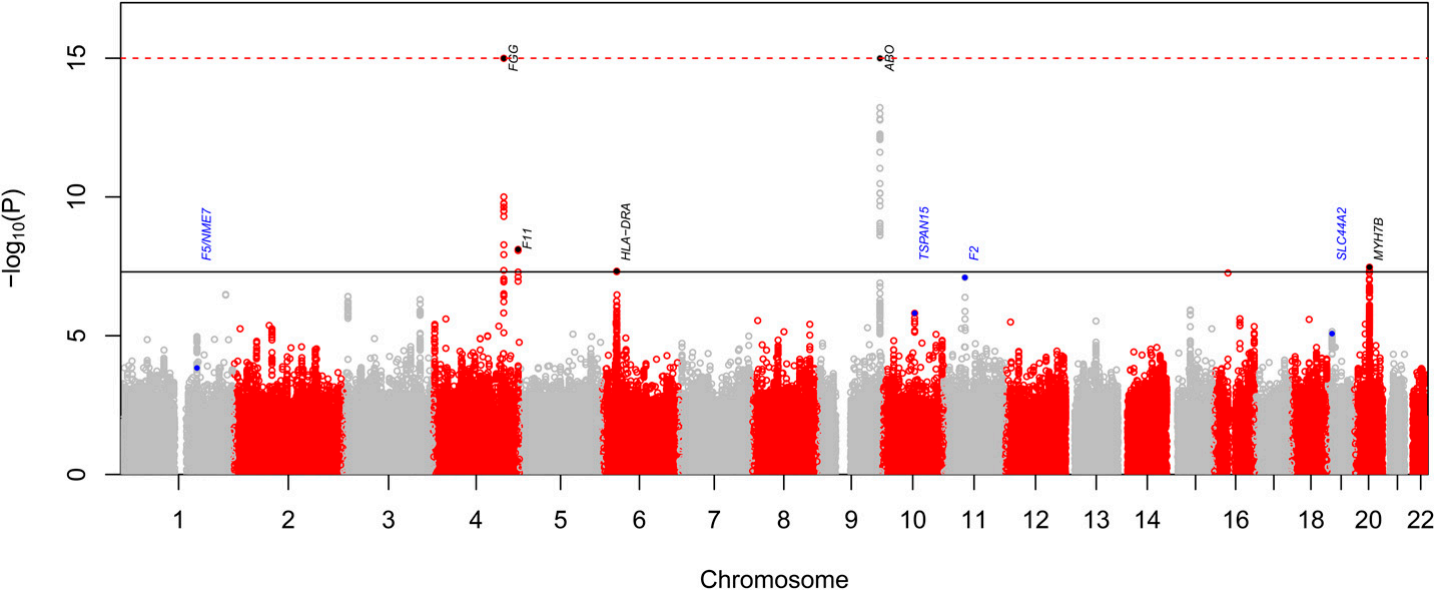
Manhattan plot of −log_10_(*P*) values derived from meta-analysis of discovery and replication cohorts. Points higher up correspond to variants more strongly associated with chronic thromboembolic pulmonary hypertension (CTEPH). Variants reaching genome-wide significance (*P*_CTEPH_ < 5 × 10^−8^) are marked in black, and variants discovered using coanalysis with pulmonary embolism are marked in blue, both labeled with the likely associated gene. The black horizontal line denotes genome-wide significance. Values of −log_10_(*P*) larger than 16 are truncated to 16.

A Manhattan plot of meta-analyzed *P* values is shown in Figure 2. Manhattan plots for the discovery and replication cohorts alone are shown in Figures E4 and E5. Two regional associations (*FGG* and *ABO*) were found at tier 1 significance, and a further association (*MYH7B*) was found at tier 2 significance. Two further regions (*F11* and *HLA-DRA*) reached tier 3 significance on the basis of meta-analysis *P* value. Results for all SNPs reaching genome-wide significance are shown in Table 1.

**Table 1.** Genome-Wide Significant Regions for Chronic Thromboembolic Pulmonary Hypertension

Chromosome	Base Pair	rsID	MAF	OR	*P* Value	*P* Value (PE)	*V* Value	Tier	Gene
9	136137106	rs687289	0.340	1.80	6.8 × 10^−27^	5.4 × 10^−35^	6.8 × 10^−27^	1	*ABO*
4	155520930	rs7659024	0.250	1.60	9.0 × 10^−17^	4.7 × 10^−14^	3.5 × 10^−23^	1	*FGG*
4	187207381	rs2289252	0.400	1.30	7.7 × 10^−9^	3.3 × 10^−16^	3.0 × 10^−15^	3	*F11*
20	33572178	rs745849	0.430	0.76	3.4 × 10^−8^	7.5 × 10^−2^	9.7 × 10^−8^	2	*MYH7B*
6	32434481	rs17202899	0.100	1.60	4.7 × 10^−8^	5.5 × 10^−1^	9.2 × 10^−7^	3	*HLA-DRA*
11	46349696	rs149903077	0.013	3.30	7.9 × 10^−8^	1.6 × 10^−12^	3.0 × 10^−14^	3	*F2*
10	71196698	rs78677622	0.130	0.70	1.5 × 10^−6^	8.2 × 10^−12^	6.0 × 10^−13^	3	*TSPAN15*
19	10742170	rs2288904	0.210	0.76	8.3 × 10^−6^	2.2 × 10^−7^	9.3 × 10^−11^	3	*SLC44A2*
1	169272453	rs796548658	0.039	1.60	1.5 × 10^−4^	3.9 × 10^−18^	7.2 × 10^−10^	3	*F5/NME7*

*Definition of abbreviations*: OR = odds ratio; MAF = minor allele frequency; PE = pulmonary embolism.

Positions shown are GRCh37, and MAF is in control subjects. Overall odds ratios are estimated from meta-analysis *P* values and overall sample sizes. *P* value and *P* value (PE) refer to chronic thromboembolic pulmonary hypertension meta-analysis *P* values and *P* values for PE (derived from a separate genome-wide association study), respectively. *V* values can be thought of as *P* values for chronic thromboembolic pulmonary hypertension adjusted to account for overall genetic similarity with PE.

### Coanalysis with DVT and PE

**Figure 3.**
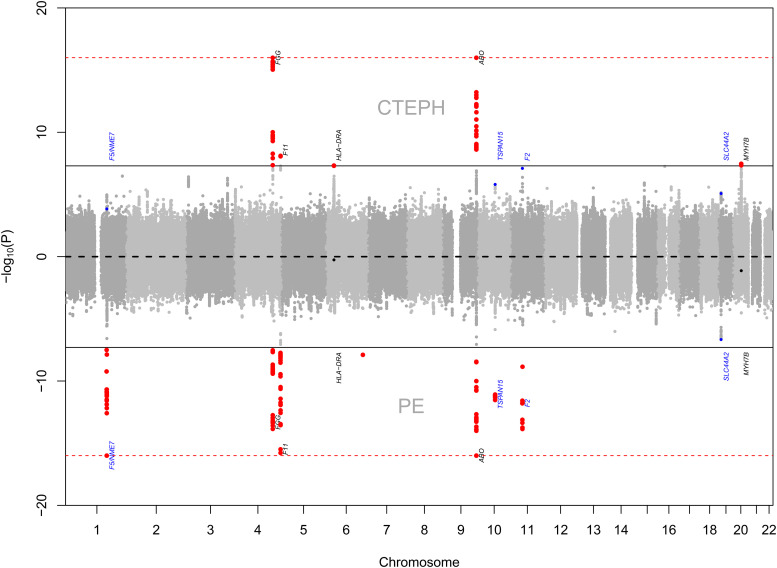
Back-to-back Manhattan plots for chronic thromboembolic pulmonary hypertension (CTEPH) and pulmonary embolism (PE). The distance from the middle line corresponds to −log_10_(*P*) values; points farther from the middle line correspond to variants more strongly associated with CTEPH (upward) or PE (downward). Values of −log_10_(*P*) larger than 16 are truncated to 16. Peak variants as in Table 1 are marked with the likely corresponding gene. There is substantial sharing between associations with CTEPH and with PE. Genome-wide associations (*P* < 5 × 10^−8^) are marked in red. Additional associations discovered through leverage (conditional false discovery rate) are marked in blue.

**Figure 4.**
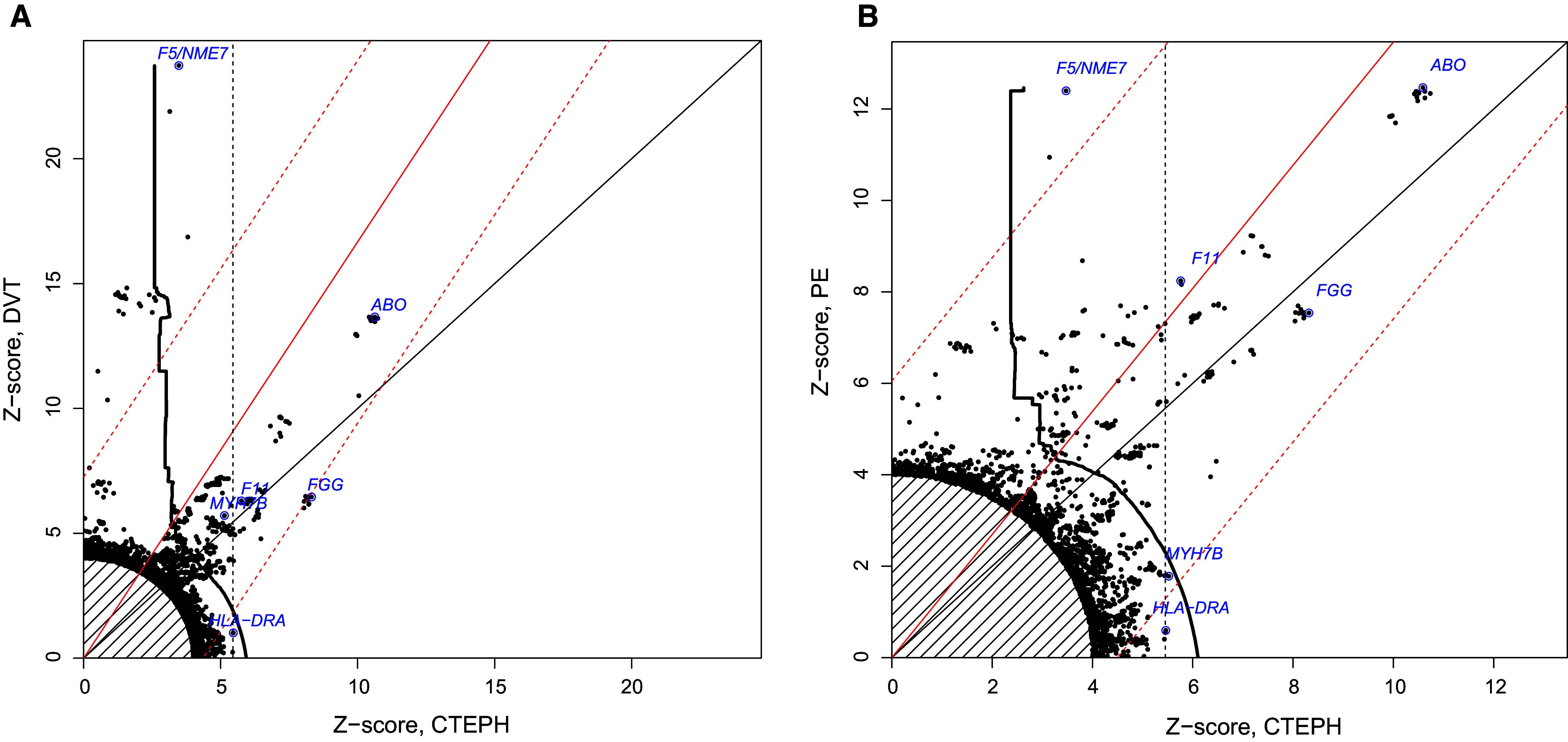
(*A* and *B*) Z-scores for chronic thromboembolic pulmonary hypertension (CTEPH) against Z-scores for deep vein thrombosis (DVT) (left) and pulmonary embolism (PE) (right). Each point corresponds to an SNP, with color and shape corresponding to chromosome as per the legend. Z-score pairs close to the origin are excluded. Points higher up correspond to variants more associated with DVT/PE, and points farther to the right correspond to variants more associated with CTEPH. Potential genes (*F11*, *F5*, *HLA-DRA*, etc.) are labeled for some SNPs. The area to the right of the dotted black line is a rejection region based on a CTEPH genome-wide significance threshold of *P*_CTEPH_ < 5 × 10^−8^. The area to the right of the solid black line is a rejection region based on the leveraged analysis using conditional false discovery rates, equivalent to a *V* value <5 × 10^−8^. The solid red line shows the expected position of Z-score pairs if SNP effect sizes for CTEPH and DVT/PE were identical. If effect sizes were identical for all SNPs, the probability of any of the points corresponding to the ∼200 SNPs reaching genome-wide significance for CTEPH or DVT/PE falling outside the dashed red lines is <0.05. We see that peak SNPs for *F5* and *HLA-DRA* fall outside the dashed lines in both plots.

The coanalyses with PE demonstrated four further associations at tier 3 genome-wide significance (*F2*, *TSPAN15*, *SLC44A2*, and *F5/NME7*). Plots of *z*-scores from the three analyses showed evidence of widespread sharing of associations with DVT and PE but differential effect sizes between phenotypes (Figures 3, 4A, and 4B).

### CTEPH GWAS Associations

#### FGG and ABO (tier 1)

We found an association with peak SNP rs7659024, approximately 4 kb downstream of the *FGG* gene. The *FGG* gene codes for the γ-chain of the fibrinogen protein, a precursor for fibrin, the principal noncellular component of blood clots. Polymorphisms in *FGG* are well known to be associated with DVT (17). The variant is also 9 kb downstream of the *FGA* gene, which codes for the α-chain of the fibrinogen complex. The strongest association (by *P* value) in CTEPH was rs687289 in the *ABO* gene, which determines ABO blood group. This locus is also known to be associated with DVT (17). Patients with non-O blood groups are at higher risk of CTEPH (18).

#### HLA-DRA, TSPAN15, F2, SLC44A2, F11 (tiers 2/3)

An association (variant rs17202899) was found at tier 2 significance in the *HLA-DRA* gene, which, to our knowledge, has not been shown to be associated with DVT or PE. The variant is strongly associated with multiple autoimmune conditions, including type 1 diabetes (19), systemic lupus erythematosus (20), and multiple sclerosis (21). Variant rs78677622, on chromosome 10, is an intron variant 10 kb upstream of *TSPAN15*, which is known to be associated with DVT (17). Variant rs149903077 on chromosome 11 is an intron variant in the *DGKZ* gene but is likely to correspond to an association of CTEPH with the *F2* gene, from which it is 390 kb upstream.

Variant rs2288904 on chromosome 19 is a missense variant in the *SLC44A2* gene, variants in which are associated with DVT (17). Variant rs2289252 on chromosome 4 is an intron in *F11*, which codes for coagulation factor 11, variants in which are associated with DVT (17). Variant rs745849 on chromosome 20 is an intron in the *MYH7B* gene, for which there are nearby associations with DVT (17), although the variant itself does not reach genome-wide significance for either DVT or PE. The variant is associated with human height and ease of tanning (13). Finally, we found an association at rs796548658 on chromosome 1 at tier 3 significance. Although the peak variant is an intron in the *NME7* gene, it is likely to represent an association of CTEPH with the *F5* gene, which is strongly associated with DVT (17). This association is notable for the relatively small effect size in CTEPH.

#### Genetic overlap with IPAH and PE

We did not find genetic evidence of shared pathology between CTEPH and IPAH. No shared genome-wide associations are evident between our findings and a recent GWAS on IPAH (6). The observed genetic correlation between IPAH and CTEPH was not significantly different from 0 (estimated *ρ_g_*, −0.37; SE, 0.38; *P* value 0.3 against H^0^: *ρ_g_*, 0) but was significantly different from 1. The genetic correlation between CTEPH and self-reported PE was significantly above zero, indicating shared genetic architecture (estimated *ρ_g_*, 1.07; SE, 0.44; *P* value 0.014 against H^0^: *ρ_g_*, 0) but not significantly different from 1, indicating that identical genetic architecture could not be ruled out with this analysis. We concluded that on the basis of genetic correlation, CTEPH is more similar to PE than to IPAH.

#### Comparison of DVT, PE, and CTEPH

We found a substantial difference in observed effect sizes of variants in the *F5* gene between DVT, PE, and CTEPH. We also noted that the *HLA-DRA* and *MYH7B* variants are not known to be associated with DVT or PE.

For both the *F5* and *HLA-DRA* regions, the probability of observing effect sizes at least as different as those seen under a null hypothesis of identical true effect sizes between CTEPH and DVT or PE was <0.05, using a Bonferroni correction over all variants reaching genome-wide significance for either disease. This is shown in Figure 2.

If the observed odds ratio of the peak SNP for *F5* in DVT (or SNPs in close linkage disequilibrium) were equal to the true odds ratio in CTEPH, our study would have had >99% power to detect an association at tier 1 significance. Likewise, if the observed odds ratio for the *HLA-DRA* association found in our study corresponded to the true effect size in DVT, then the study on DVT would have >99% power to detect the association.

We conclude that the effect size of causal variants in *F5* and *HLA-DRA* in CTEPH is different from the effect of those variants in DVT and in PE. We cannot conclude that the effect size of the causal variant in *MYH7B* differs between CTEPH and DVT or PE.

## Discussion

We report the first GWAS in CTEPH, comprising a multinational study on a cohort with sufficient power to find common-variant associations of reasonable size. In general, the associations we find are consistent with a shared genetic association of venous thromboembolism, although we identify important differences in genetic architecture from PE and DVT. CTEPH is a partially heritable polygenic disease. It does not develop randomly among patients with pulmonary emboli, nor is development of CTEPH governed entirely by environmental triggers. If this were the case, all genetic associations for both diseases would have identical size (and variants in *F5* and *HLA-DRA* do not). Historical debate has for decades posited that the similarity in pathophysiology, presence of thrombus in some cases of IPAH, and absence of index PE in up to one-fourth of cases of CTEPH suggest that CTEPH is not simply the consequence of disordered thrombus fibrinolysis but instead is a potential overlap of distal cases of CTEPH and IPAH (22). Our work supports evidence that CTEPH and IPAH are distinct and that, despite similar vascular remodeling, inflammation, and involvement of dysregulated angiogenesis, the underlying etiologies are different. This is consistent with work examining CTEPH cohort demographics and phenotypes (23). Genetic associations of underlying susceptibility to vascular remodeling or pulmonary hypertension do not appear to be major drivers of CTEPH in this study.

The smaller effect sizes of variants in *F5* in CTEPH may be an example of index-event bias (24), a phenomenon in which the effect of a risk factor is underestimated due to the dominance of other factors. Specifically, the large effect of the *F5* Leiden variant in causing thromboembolic disease may paradoxically mean that patients with PE carrying an *F5* Leiden variant have a lower burden of other genetic and environmental risk factors for CTEPH and are hence less likely to develop CTEPH after PE than those without the variant. This could also account for the apparently smaller relative effect of *F5* variants in PE than in DVT seen in Figures 4A and 4B.

To our knowledge, *HLA-DRA* has not previously been shown to be associated with either DVT or PE, although variants in the locus have been associated with a range of immune-related phenotypes (25), likely reflecting a role in the processing and presentation of major histocompatibility complex molecules. Increased CTEPH risk has long been linked with underlying autoimmune and hematological disorders (4). In addition, a variety of inflammatory cytokines are elevated in CTEPH and correlate with pulmonary artery inflammatory cell infiltration and CTEPH severity (26).

An important shortcoming of our work is imperfect geographic matching between cases and control subjects in the replication cohort, resulting in a degree of inflation in summary statistics. This is unavoidable with our current dataset. To manage this, we forcibly rescaled χ^2^ statistics to remove the inflation (*see* the Methods section of the online supplement). We also have not adjusted for age and sex, although the need to do this in a disease with an incidence of less than 1.0% has previously been demonstrated to be minimal. Our study is also not powered to perform subanalyses of patients with and without known venous thromboembolism, and future work should consider this. Reassuringly, our overall findings are not unexpected. A further shortcoming of our work is its restriction to individuals of Western or Central European ancestry, and further investigation into the genetic architecture of CTEPH in other ethnicities is warranted. Our study also cannot shed any light on the contribution of rare genetic variants to pathophysiology.

In summary, we provide the first large-scale GWAS in this rare disease, and we demonstrate, for the first time, to our knowledge, the genetic architecture of a complex condition leveraged against comparator datasets. These analyses establish the primacy of dysregulated thrombosis/fibrinolysis in etiology and extend our understanding of the possible contribution of additional pathophysiological mechanisms, including inflammation. CTEPH did not share any genetic associations with IPAH further confirming that despite significant shared pathophysiology, these conditions have divergent etiology.
